# Evaluation of Synthetic Peptides from *Schistosoma mansoni* ATP Diphosphohydrolase 1: In Silico Approaches for Characterization and Prospective Application in Diagnosis of Schistosomiasis

**DOI:** 10.1021/acsinfecdis.4c00697

**Published:** 2025-01-14

**Authors:** Danielle Gomes Marconato, Beatriz Paiva Nogueira, Vinícius Carius de Souza, Rafaella Fortini Grenfell e Queiroz, Clovis R. Nakaie, Eveline Gomes Vasconcelos, Priscila de Faria Pinto

**Affiliations:** † Department of Biochemistry, Institute of Biological Sciences, Federal University of Juiz de Fora, Juiz de Fora, Minas Gerais 36036-900, Brazil; ‡ Laboratory of Applied Toxinology (LTA), Butantan Institute, São Paulo 05503-900, Brazil; § Laboratory of Diagnosis and Therapy of Infectious Diseases and Cancer—Fiocruz DATA., René Rachou Research Center, Oswaldo Cruz Foundation, FIOCRUZ, Belo Horizonte, Minas Gerais 30190-002, Brazil; ∥ Department of Biophysics, Paulista School of Medicine, Federal University of São Paulo, São Paulo 04044-023, Brazil

**Keywords:** schistosomiasis, SmATPDase1, peptides, immunogenicity, diagnosis

## Abstract

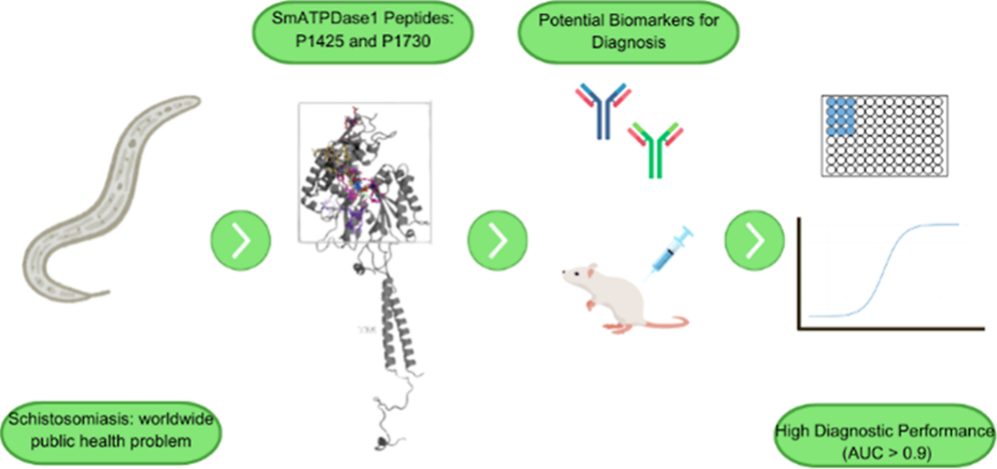

Schistosomiasis is the infection caused by *Schistosoma mansoni* and constitutes a worldwide public health problem. The parasitological recommended method and serological methods can be used for the detection of eggs and antibodies, respectively. However, both have limitations, especially in low endemicity areas. Thus, new approaches for the diagnosis of schistosomiasis are essential. In this study, a six-amino acid peptide and derived sequences from SmATPDase1 were synthesized for the evaluation of immunogenicity. SmATPDase1 is included in a protein group in *S. mansoni* tegument; therefore, its peptides could be potential candidates for diagnostic antigens. In the hypothetical SmATPDase1 three-dimensional structure, peptides are located in a region exposed and accessible to antibody binding. In addition, peptide amino acid sequences are conserved in the most relevant *Schistosoma* species and have low identity with human NTPDases isoforms. Swiss mice immunization resulted in significant anti-peptide polyclonal antibodies production, which recognized a 63 kDa protein in tegument and adult worm preparations. By immunofluorescence microscopy, polyclonal antibodies also identified this enzyme in cercariae. Sera of infected animals presented high seropositivity in ELISA-peptides, with an area under curve (AUC) greater than 0.96 for all peptides. In mice with low parasite burden, we observed a seropositivity AUC > 0.9. Reactivity in the prepatent period exhibited AUC values greater than 0.94 for all peptides. Anti-P1425 monoclonal antibodies were successfully produced, and mAbs recognized the integral protein in ELISA and Western blots. The data indicate that peptides from SmATPDase1 are potential biomarkers for schistosomiasis, and anti-peptide antibodies are interesting tools for the detection of the infection.

Schistosomiasis is the infection caused by flatworms of the genus *Schistosoma*.^1^ It is estimated that about 250 million people are affected in 78 countries around the world, which prevails as a worldwide public health problem.^1,2^ The main species responsible for infection are *Schistosoma mansoni*, *Schistosoma japonicum,* and *Schistosoma hematobium*.^1,2^ In America, *S. mansoni* is the infectious agent of the disease.

Many approaches are available for the diagnosis of schistosomiasis. The method recommended by the World Health Organization for screening infections at the populational level is based on microscopic analysis of eggs in feces (*S. mansoni*, *S. japonicum*) or urine (*S. hematobium*).^1,2^ However, parasitological examination has limitations, mainly in low endemic areas.^3−5^

Indirect methods have an advantage over microscopic techniques since they are more sensitive but have low specificity, i.e., reduced ability to discriminate between active and past infections.^6^ In search of molecules to improve specificity, some studies concentrate on exploring specific biomarkers.^7,8^

The *S. mansoni* tegument is a direct interface between *S. mansoni* and the bloodstream of the host and is thus rich in target proteins for application in diagnosis, vaccines, and treatment.^9,10^ Based on this, NTPDases (EC 3.6.1.5) exhibit a significant role.^11,12^ These enzymes activated by Ca^2+^ or Mg^2+^ hydrolyze di- and triphosphate nucleosides.^13,14^ In mammals, eight NTPDases isoforms were identified, and they are associated with many biological processes such as cellular proliferation, contraction, apoptosis, coagulation, and others.^13,14^

Related enzymes are described in different organisms, including parasites.^15,16^ In *S. mansoni*, two isoforms were characterized.^17−20^ Also known as ATP diphosphohydrolases or SmATPDases, they represent an important part of proteins in helminth tegument.^10^

In this work, the immunogenicity of synthetic peptides belonging to the *S. mansoni* SmATPDase1 was in silico and in vivo evaluated to verify its potential application in schistosomiasis diagnosis.

## Results

### In Silico Analyses of Peptides from *S. mansoni* ATP Diphosphohydrolase 1

**Figure 1:**
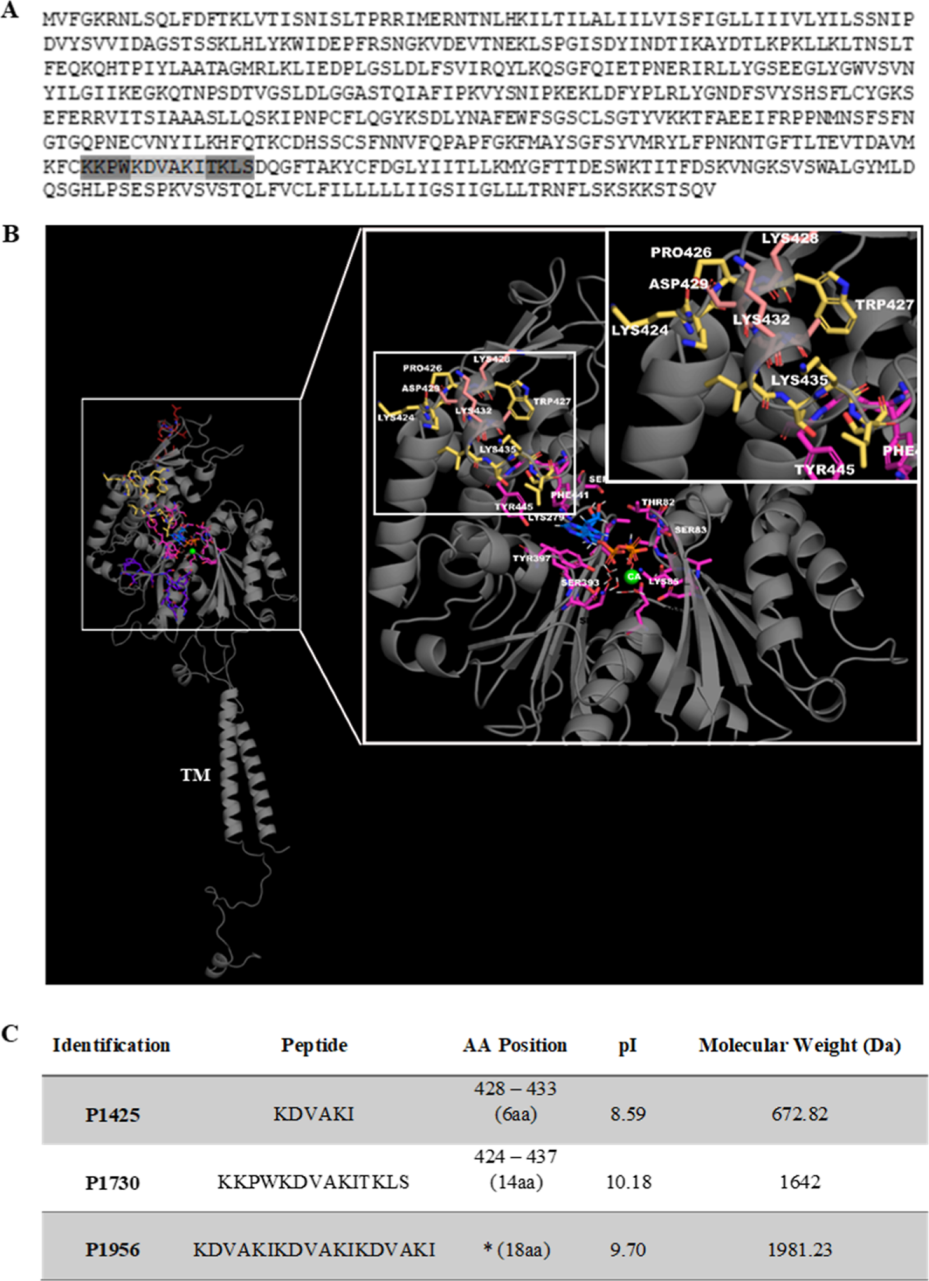
Localization and characterization of P1425 e P1730 peptides from *Schistosoma mansoni* ATPDase1. (A) Primary structure of *S. mansoni* ATPDase1 (Uniprot; ID, Q7YTA4), showing amino acid residues of the peptide P1730 (14 aa) in gray and highlighting residues of the peptide P1425 (6 aa) in light gray. *The peptide P1956 (18 aa) was obtained by the synthesis of the P1425 fragment in triplicate. (B) Three-dimensional structure of SmATPDase1 showing the peptides P1425 and P1730 in light pink and yellow, respectively. Molecular docking shows the active site in purple, substrate analogue (ANP) in blue, and cofactor Ca^2+^ in green. TM, transmembrane domain. (C) Physicochemical characteristics; AA and aa, amino acids; pI, isoelectric point.

Three peptides, P1425, P1730, and P1956, were designed from the primary structure of *S. mansoni* ATP diphosphohydrolase1, also known as SmATPDase1. The enzyme consists of 544 amino acid residues, and its integral sequence and regions corresponding to peptides P1730 and P1425 are shown in Figure 1A. The peptide P1956 corresponds to the sequence KDVAKI (P1425) in triplicate.

The hypothetical three-dimensional structure of SmATPDase1, created by comparative modeling, shows the amino acid residues corresponding to P1430 and 1730 peptides in light pink and yellow, respectively, exposed and possibly available for antibody binding (Figure 1B). The interaction between the active site (lilac), ANP substrate (ADP analogue), and the cofactor Ca^2+^ is also represented (Figure 1B). A summary of the main characteristics of the peptides such as amino acid position, theoretical mass, and isoelectric point (pI) is shown in Figure 1C. Instability index classified the sequences as stable molecules.

**Figure 2:**
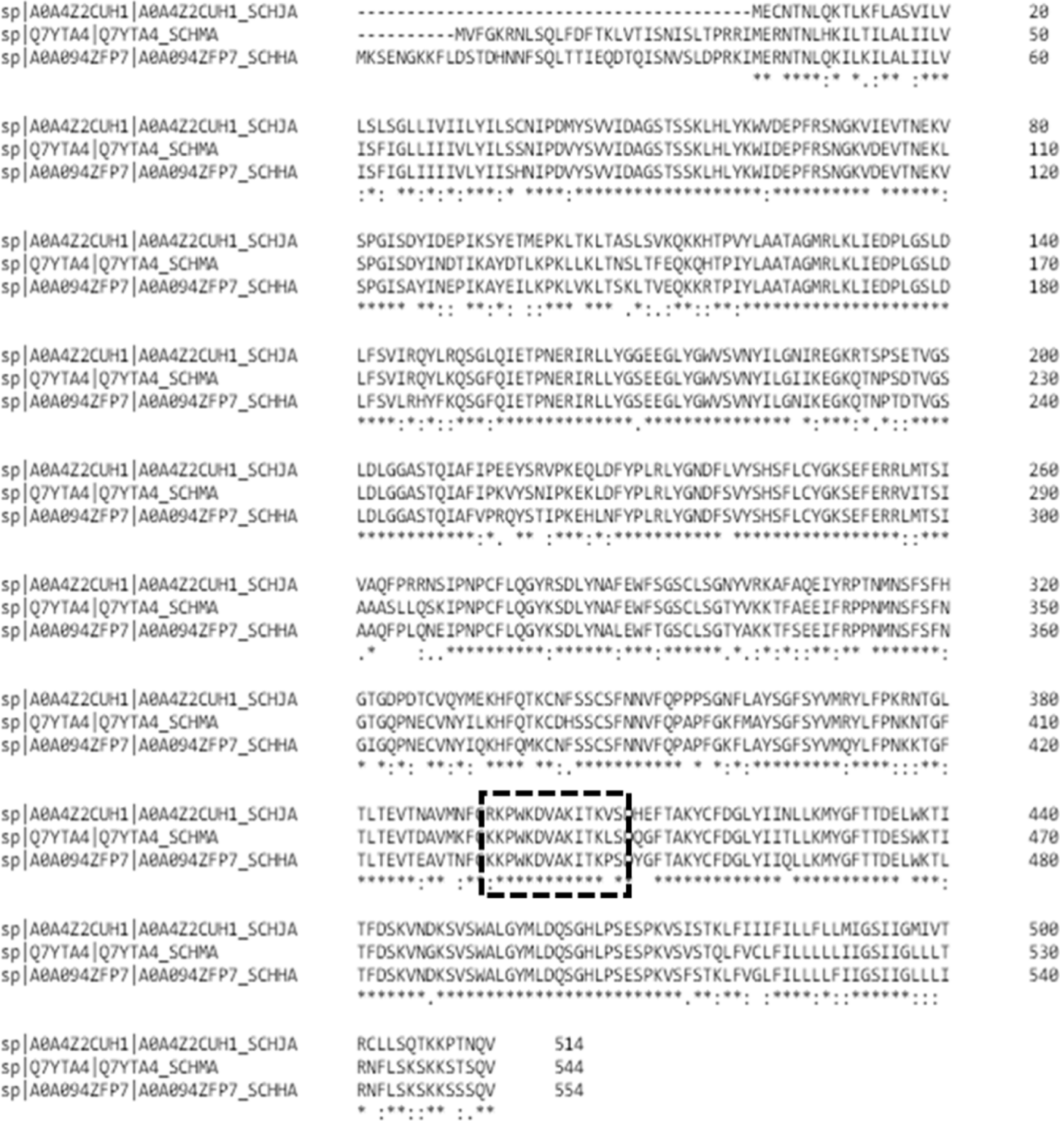
Alignment of ATP diphosphohydrolases from *S. mansoni*, *S. hematobium,* and *S. japonicum*. Multiple alignment of primary sequences from *S. mansoni* (ID, Q7YTA4), *S. hematobium* (ID, A0A094ZFP7), and *S. japonicum* (ID, A0A4Z2CUH1) using Clustal Omega software. The amino acid sequences of peptides P1425 and P1730 were highlighted in the black rectangle. * Identity.

The peptide P1730 sequence analysis using BLAST in the UniProt database identified putatives ecto-NTPDase from *S. hematobium*, *S. japonicum,* and *Schistosoma margrebowiei* and three other unrelated proteins with lower similarity, possibly due to the peptide size. Primary structure alignment of NTPDases from species with clinical relevance—*S. mansoni*, *S. hematobium,* and *S. japonicum*—showed high identity among them, P1730 (92.3%) and P1425 (100%) (Figure 2).

**Figure 3:**
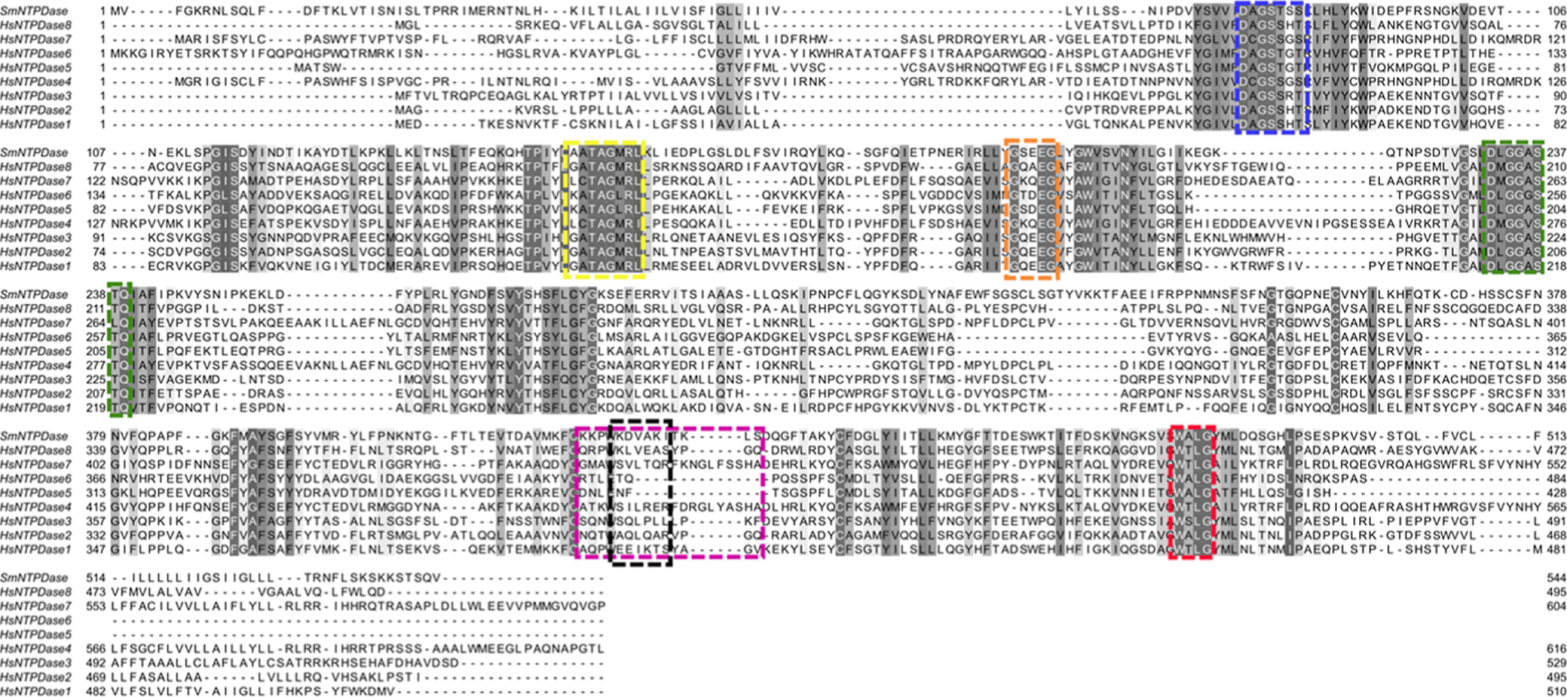
Multiple alignment of SmATPDase1 and human NTPDases 1–8. Primary sequences from eight human NTPDases isoforms were aligned with the helminthic enzyme, using Clustal Omega software. The regions containing the peptides P1425 and P1730 were highlighted in black and purple, respectively. Identity and similarity are represented by different degrees of gray. The catalytic site is represented by Apyrase Conserved Regions (ACRs) 1 to 5: blue, yellow, orange, green, and red, respectively. Hs, *Homo sapiens*; Sm, *S. mansoni*; SmATPDase (ID, Q7YTA4); NTPDase1 (ID, P49961); NTPDase2 (ID, Q9Y5L3); NTPDase3 (ID, O75355); NTPDase4 (ID, Q9Y227); NTPDase5 (ID, O75356); NTPDase6 (ID, O75354); NTPDase7 (ID, Q9NQ27); NTPDase8 (ID, Q5MY95).

In addition, no similarity was found when amino acid sequences of peptides P1425 (black) and P1730 (lilac) were compared with those of mammalian NTPDases 1–8, and they are not included in the characteristic conserved domains (ACRs; Apyrase Conserved Regions) of the NTPDase family, which form the active site (Figure 3).

Epitopes’ prediction constitutes a criterion for evaluating a good candidate for diagnosis. Thus, prediction of a B-cell epitope by the ABCPred server, using SmATPDase1 sequence and selected threshold >0.75, showed the KDVAKI presence in many sequences. As shown in Table 1, among others, one 14-mer length epitope containing peptide P1730 and almost the entire sequence of the peptide P1425 were predicted to be epitopes (Table 1). In addition, the peptide P1730 (KKPWKDVITKLS) has potential ability to bind MHC class II, with an IC_50_ of 40.90 nM (Table 1).

**Table 1:** Best B-Cell and T-Cell Epitopes Related to Peptides P1425 and P1730

	sequence	amino acid length	threshold
B-cell epitope	CKKPWKDVAKITKL	14	0.75
T-cell epitope	KKPWKDVAKITKLS	14	IC50 < 50

### Immunogenicity and Application of Anti-Peptide Antibodies in SmATPDase1 Localization

**Figure 4:**
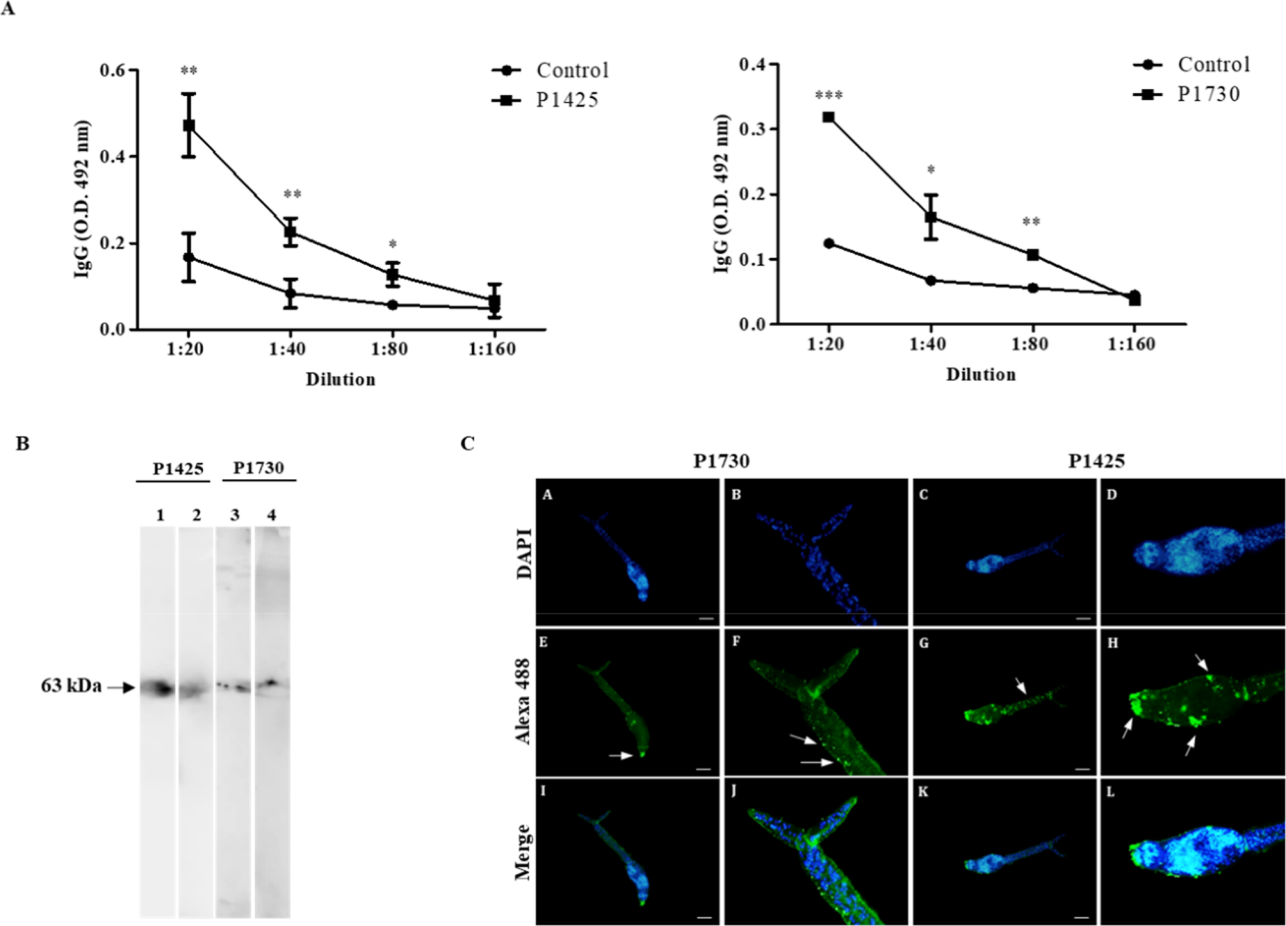
Immunostimulatory properties of peptides P1425 and P1730 and identification and immunolocalization of SmATPDase1. (A) Microplates sensitized with 1 μg/mL of the synthetic peptides were incubated with sera from immunized female Swiss mice at dilutions 1:20 to 1:160. Reactivity was represented by optical density (OD) and data expressed as mean ± standard deviation (SD) and analyzed by Student’s *t*-test. ***p ≤ 0.001; **p ≤ 0.01; *p ≤ 0.05. (B) Anti-peptide polyclonal antibodies (1:100) recognize SmATPDase1 in *S. mansoni* tegument (1; 3) and adult worms’ homogenate (2; 4) by Western blots and (C) immunolocalize this protein in cercariae by fluorescence microscopy. (A–D) Nuclear staining by DAPI; (E and F) anti-P1730 polyclonal antibodies; (G and H) anti-P1425 polyclonal antibodies; (F and H) zoom and (I–L) merge of the respective images. Bars: 50 μm.

After immunization, ELISA assay was performed to verify the production of anti-peptide polyclonal antibodies (Figure 4A). Significant IgG antibody production was observed for peptides P1425 and P1730, up to a 1:80 dilution (Figure 4A). Therefore, both peptides have the ability to stimulate antibodies producing B-cells, corroborating the in silico analysis.

The polyclonal antibodies produced against peptides P1425 and P1730 identified SmATPDase1 in the tegument and adult worm homogenate from *S. mansoni*, recognizing a band of approximately 63 kDa, as demonstrated by Western blot (Figure 4B). In addition, specific signals were evidenced in *S. mansoni* cercariae by immunofluorescence microscopy, as revealed by anti-IgG antibodies coupled to Alexa Fluor 488 (Figure 4C). These data confirm the potential application of these anti-peptide polyclonal antibodies for functional studies of the parasite SmATPDase1.

### Potential Application of Peptides from SmATPDase1 in Diagnosis of Schistosomiasis

**Figure 5:**
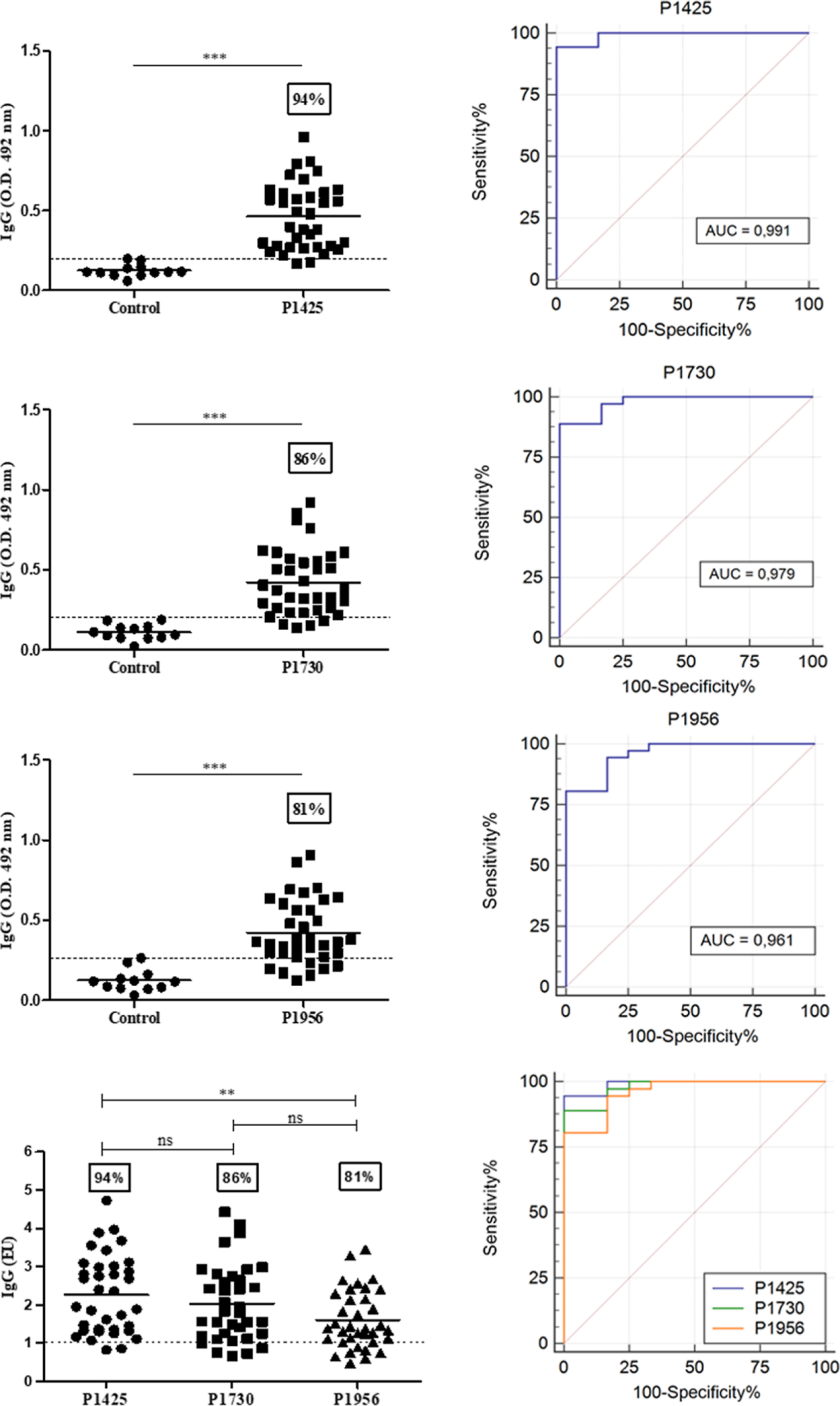
Reactivity of *S. mansoni*-infected mice sera and peptides P1425, P1730, and P1956. Individual reactivity of sera from healthy or infected Swiss mice at dilution 1:200 (left column), and specificity and sensitivity verified by receiver operating characteristic (ROC) curves and area under curve (AUC), in which AUC >0.9 indicates high test performance (right column). Data are presented as OD or ELISA units (EU). The dotted line represents cutoff values (mean of the control group plus twice the SD). The statistical significance of differences was determined by the Mann–Whitney or Kruskal–Wallis test. ***p ≤ 0.001; **p ≤ 0.01; *p ≤ 0.05; ns, not significant.

The reactivity of sera from *S. mansoni*-infected mice against peptides P1425, P1730, or P1956 was evaluated by ELISA, and significant difference (*p* < 0.001) was observed when compared to healthy animals, used as a control group (Figure 5). The seropositivity for P1425, P1730, or P1956 was 94%, 86%, and 81%, respectively, being similar between P1425 and P1730 and significantly (*p* < 0.01) higher for P1425 compared to that for P1956 (Figure 5). In addition, ROC curves were constructed to check the ability to discriminate between uninfected and infected mice (Figure 5). All peptides exhibited AUC values higher than 0.95, indicating high sensibility and specificity. For seropositivity, the last parameter, all provided a value of 100% (Figure 5).

**Figure 6:**
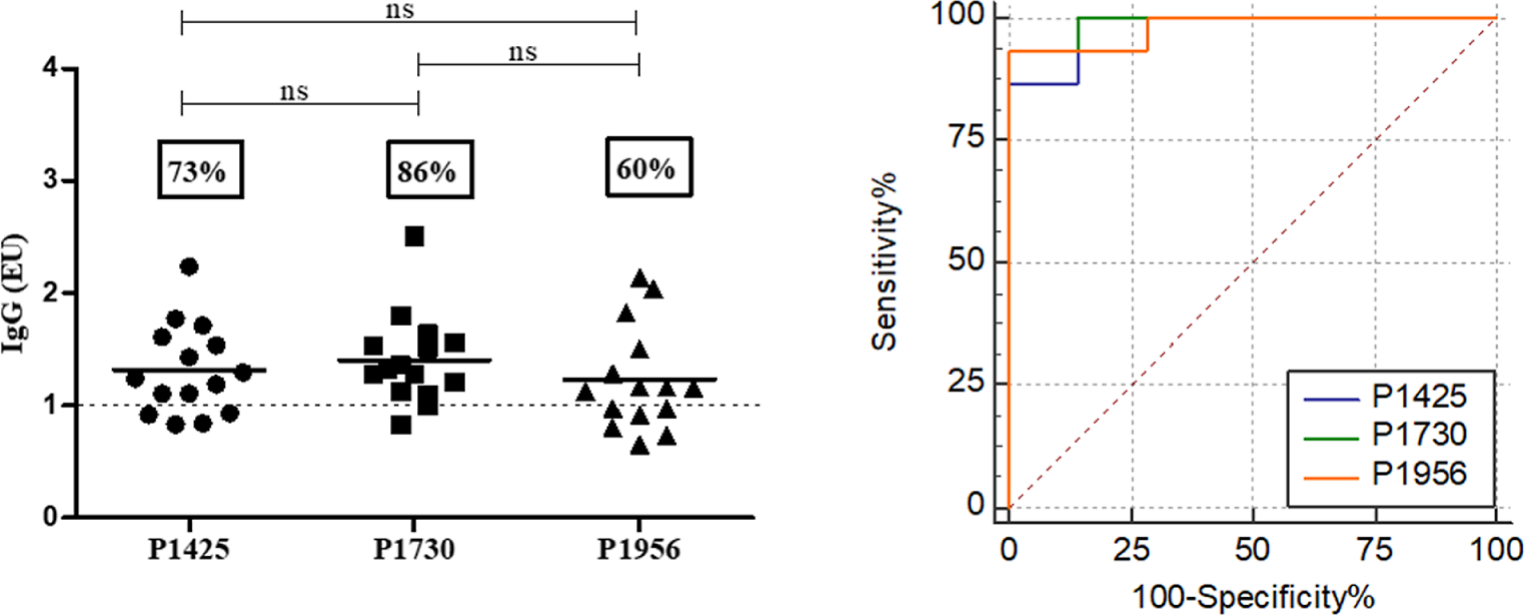
Reactivity of sera from infected mice with low parasite load. Sera from mice infected with 25 cercarias (dilution 1:200) were evaluated against peptides P1425, P1730, and P1956 by ELISA (A). The specificity and sensitivity of the assay were verified by the AUC-ROC curve. Data are presented as EU. The dotted line represents cutoff values (mean of the control group plus twice the SD). The statistical significance of differences was determined by the Kruskal–Wallis test. ns, not significant.

**Figure 7:**
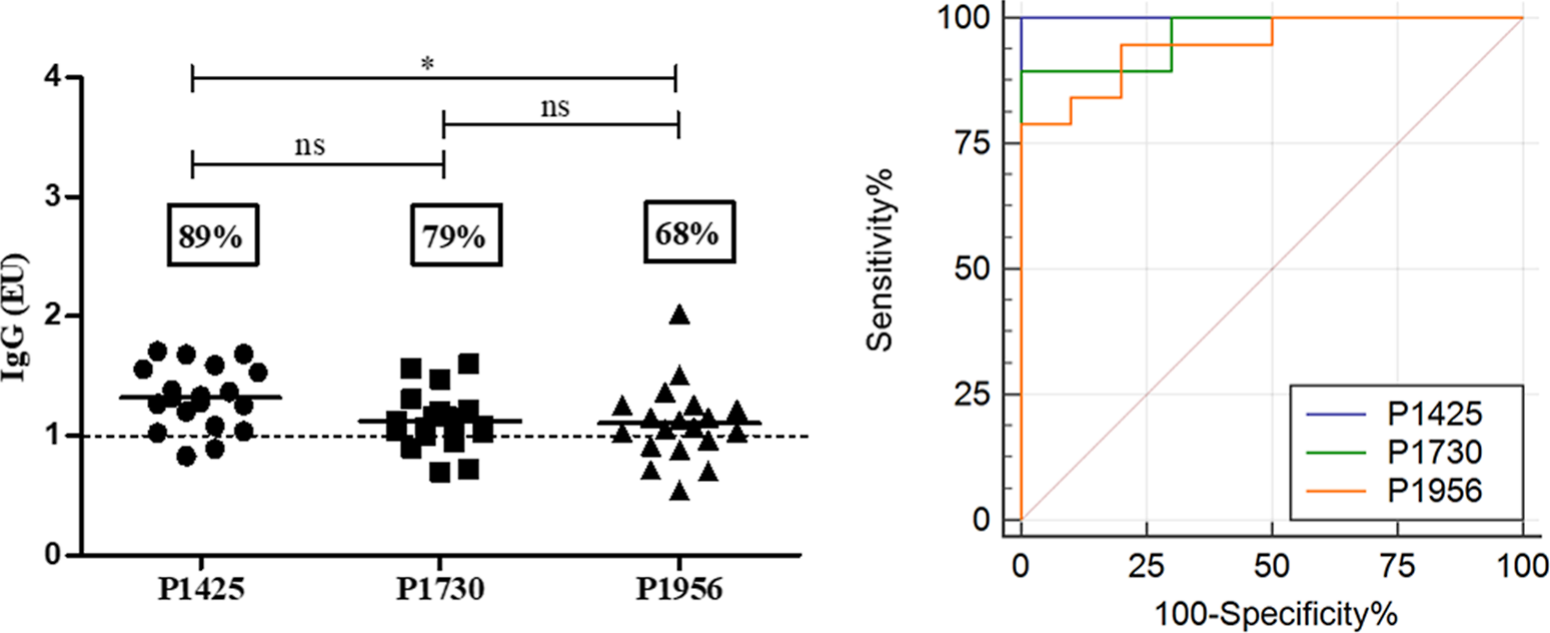
Reactivity of sera from infected mice in the prepatent period. Sera from infected mice (1:200) on the 10th to 25th days post-infection were evaluated against peptides P1425, P1730, and P1956 by ELISA. The specificity and sensitivity of the assay were verified by the AUC-ROC curve. Data are presented as EU. The dotted line represents cutoff values (mean of the control group plus twice the SD). The statistical significance of differences was determined by the Kruskal–Wallis test. *p ≤ 0.05; ns, not significant.

The seropositivity of peptide-ELISA was also studied in infected mice with a low parasitic load (Figure 6) or in the prepatent period (Figure 7). The seropositivity in the first assay was 73%, 86%, or 60% for peptides P1425, P1730, and P1956, respectively, and specificity was 100% for P1425 and P1730, with AUC > 0.97 for all of them (Figure 6). In addition, seropositivity of the mice group in the prepatent period was significantly elevated for all peptides (>68%), showing a specific reactivity with AUC values greater than 0.94 (Figure 7).

**Figure 8:**
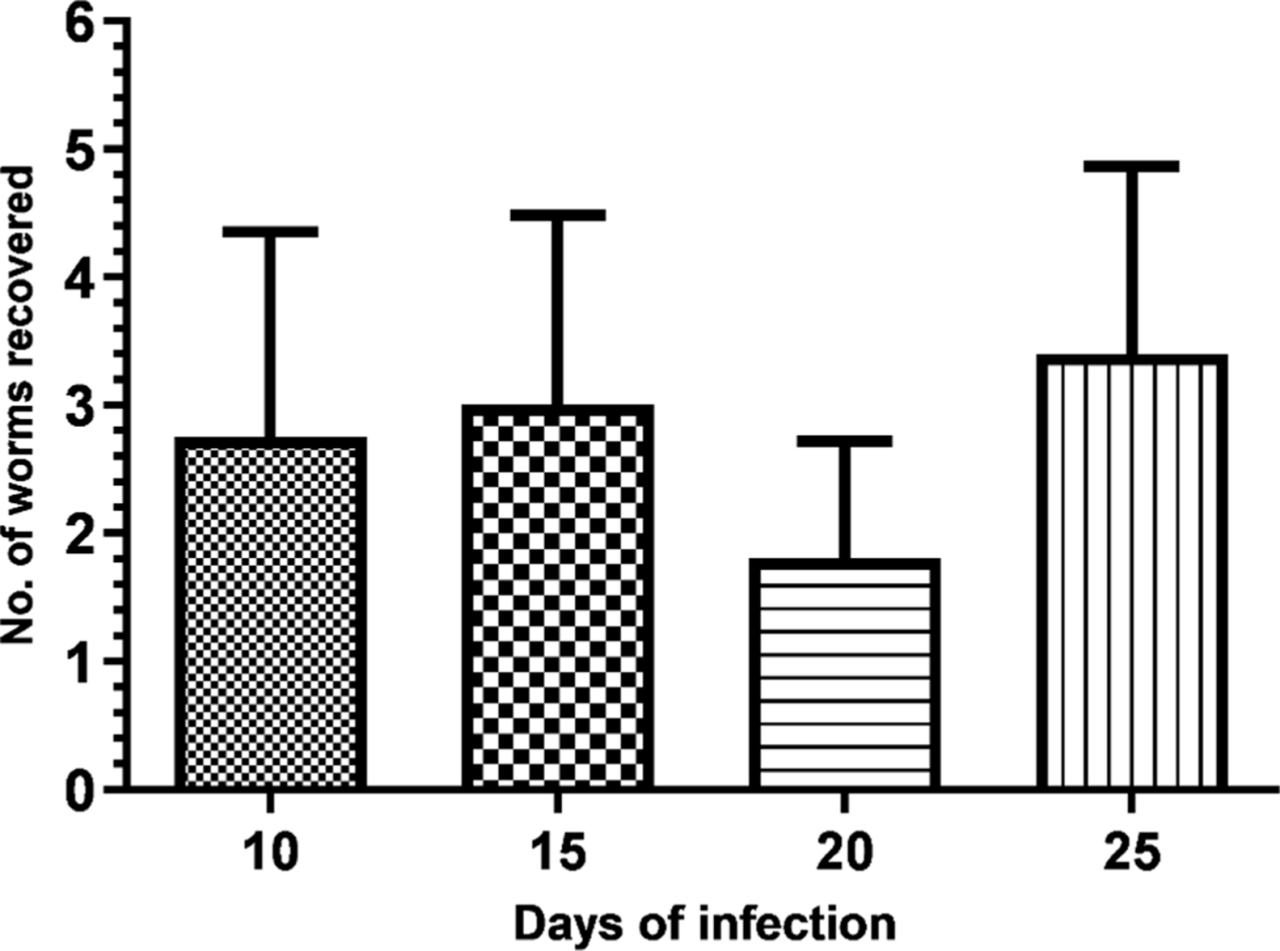
Recovery of adult worms and evaluation of parasitic load in animals infected with *S. mansoni*: at the end of 55 days of infection, the mice (5 per group) that had their sera collected from the 10th to the 25th day post-infection were euthanized and perfused for adult worm counting.

To verify that the animals were indeed infected, perfusion and counting of adult *S. mansoni* worms were performed. The number of recovered worms is shown in Figure 8. Observing the data, the groups presented animals with active infection and low parasitic load, as expected after infection with 25 cercariae. With these results, the peptides demonstrate the ability to detect the disease even before egg-laying, an important factor for disease control.

### Evaluation of Anti-Peptide Monoclonal Antibodies

**Figure 9:**
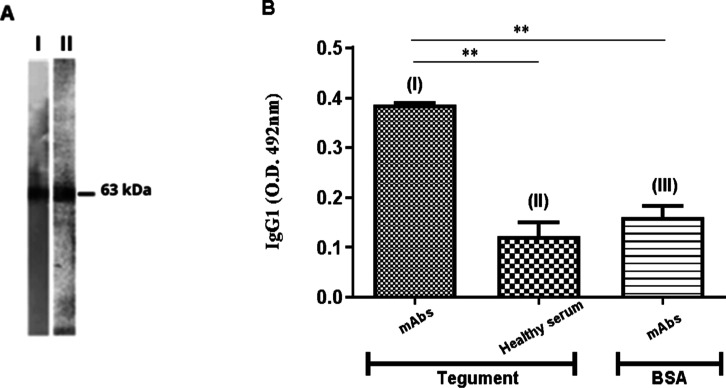
Anti-peptide IgG1 mAbs recognize *S. mansoni*SmATPDase 1. (A) Aliquot (100 μg of total protein) of SWAP (I, soluble adult worm antigen) or worm’s tegument (II) preparation was submitted to electrophoresis in 10% SDS-PAGE, electroblotted onto the nitrocellulose membrane, and the Western blots were developed with mAbs, as revealed by chemiluminescence. (B) Reactivity of mAbs or “pooled” sera from C57BL/6 healthy mice was quantified by ELISA using worm’s tegument (I and II) or BSA (III) as the coating antigen. The result is represented by the mean ± SD of OD. The statistical significance of differences was determined using Student’s *t*-test. **p < 0.01.

Monoclonal IgG1 antibodies were produced against the peptide P1425, which recognized only a 63 kDa band in SWAP (I) and adult worm’s tegument (II) by Western blots (Figure 9A). Their reactivity with worm’s tegument (I; 0.385 ± 0.004) was significantly higher than that observed with Bovine Serum Albumin (BSA) (III; 0.161 ± 0.022; *p* < 0.01), both used as the coating antigen in ELISA, or when compared to the reactivity of sera samples from the healthy mice group with worm’s tegument (Figure 9B; II; 0.122 ± 0.003).

## Discussion

In a previous study, the six-amino acid peptide named KDVAKI or P1425 from SmATPDase1 reacted with IgE and IgG1 immunoglobulins of sera from *S. mansoni*-infected mice, indicating it as a relevant epitope.^35^ The identification and characterization of antigenic epitopes are important to understand the immune response against infections and for the development of vaccines and immunodiagnostic methods.^36^ Thus, our aim in this study was to verify other characteristics of this peptide and explore its potential application as an immunodiagnostic marker. SmATPDase1 shares conserved regions with NTPDases of other parasites,^37,38^ which justify the choice of peptides, instead of the integral protein, for a specific diagnosis.

Besides P1425, two additional peptides from *S. mansoni* were synthesized based on the primary structural sequence of *S. mansoni* SmATPDase1. The first is a 14-mer length fragment, designated P1730, and the second one is a triple repetition of P1425, named P1956. Peptide synthesis with epitope repeats favors exposure and availability for antibody binding, as indicated by Yagi et al.^39^ that showed synthetic peptides from a repetitive sequence of a *Plasmodium falciparum* protein inducing antibody response greater than other regions selected.

Currently, the bioinformatics approach has been used as a tool to improve diagnosis methods and vaccines against schistosomiasis.^7,8,40,41^ In light of this, the first step was an in silico analysis to verify whether the peptides fit some criteria to be a good candidate for diagnosis. Initially, according to a hypothetical three-dimensional structure, amino acid residues composing peptides P1730 and P1425 were located on the extracellular domain of SmATPase1, available to recognition by the host immune system and antibody binding, criteria also evaluated by Carvalho et al.^7^ In addition, they do not belong to the enzyme catalytic site, in which the ACRs (Apyrase Conserved Regions) are gathered, a characteristic shared by the NTPDase family.^13,37^

Interestingly, peptides P1425 and P1730 amino acid sequences are highly conserved in three of the most relevant *Schistosoma* species, and their applications could be extended not only in areas affected exclusively by *S. mansoni* but also in Africa and Asia countries.^1,2,6^

In the next step, homology between peptides and human NTPDases isoforms was evaluated, an important stage to search for specific therapeutic and diagnostic targets. SmATPDase 1 has several characteristics shared with human NTPDases, like the presence of two transmembrane domains anchoring surface-located extracellular domains.^13,14^ In an exquisite way, no match was found between the corresponding peptide region in the parasite enzyme and eight human NTPDases isoforms, reducing the probability and risks of cross-reactivity in the host.

In addition, peptides P1425 and P1730 belonging to SmATPDase1 were predicted as B-cell and T-cell epitopes. Moreover, an IC_50_ value for a good bind to MHC class II is about 500 nM,^42^ ten times higher than the one found (IC_50_ 40.90 nM), confirming the potential immunogenicity of peptides P1425 and P1730.

In fact, experimental procedures confirmed the in silico analyses. After three steps of immunization, polyclonal antibodies reacted with P1425 and P1730. It is relevant to note that different factors can interfere with the successful induction of immune response, including peptide size, adjuvant, and number of immunizations,^36^ and different protocols can be further developed aiming to increase anti-peptides’ antibodies’ titers.

SmATPDases are one of the most abundant proteins in *S. mansoni* tegument,^10^ and they are possibly involved with important functions on the parasite adult stage.^11,12^ Here, Western blots developed with anti-peptides polyclonal antibodies recognized a protein of 63 kDa in adult worm homogenate and tegument, which corresponds to SmATPdase1 molecular weight.^17,18,43^ These results confirm the studies carried out by Emídio et al.,^35^ in which a 63 kDa protein was immunoprecipitated by anti-P1425 antibodies and also recognized by anti-CD39 (human NTPDase1) antibodies, suggesting the presence of an NTPDase isoform. Subsequently, SmATPDase1 identity was confirmed by mass spectrometry of this reactive protein contained in electrophoresis gel.^35^

In addition, antibodies against peptides P1425 and P1730 identified the SmATPDase1 in cercariae, the infective stage for humans.^1,2^ This finding supports previous studies that demonstrate SmATPDase expression at different parasite developmental stages,^17−20^ other important criteria for a good candidate for diagnosis.^7^ Our research group has successfully used anti-peptide antibodies as tools to study parasite NTPDases.^44−46^ Now, we show here new anti-peptide antibodies for specific studies of *S. mansoni* SmATPDase1, an enzyme shared with other *Schistosoma* species and still poorly explored. The recombinant form of this protein was first described in 2003,^18^ and since then, few studies have addressed its function in the parasite biology and its role in the course of the disease. All taken together, our results indicate a potential application of peptides P1425 and P1730 and antibodies produced against them in functional studies of *S. mansoni* SmATPDase1.

Peptides derived from SmATPDase1 were also analyzed for their antigenicity and potential application in the diagnosis of schistosomiasis. Analysis of smaller structures is interesting since some antigens used in serological techniques, such as soluble egg antigen and soluble adult worm antigen preparation (SWAP), present limitations and wide accuracy variation.^6,47^ As shown here, in general, all peptides presented in vivo have good accuracy, with an AUC value greater than 0.95. The peptide P1425 presented the best performance (AUC > 0.99) in ELISA assay using infected Swiss mice. The infection with 25 cercariae promoted a low recovery of adult worms, as expected. Just like that, anti-peptides polyclonal antibodies still were detected in sera from infected mice, with significant sensitivity and specificity (AUC > 0.97). Interestingly, all three peptides used during the prepatent stage of the schistosomiasis infection were identified by IgG antibodies (AUC > 0.94). As previously pointed out, the presence of SmATPDase1 at all stages of development may favor early diagnosis. In the period known as prepatent, i.e., interval when the infection occurred but eggs are not detected,^1^ conventional methods can lead to false negatives.^6^ Oyeyemi et al.,^48^ using an ELISA-schistosomula crude antigen, were successful in diagnosing patients from a Brazilian low endemic region. Similarly, Grenfel et al.^49^ showed high accuracy in immunoassay with a schistosomula tegument antigen and infected-mice sera from seventh and 15th dpi. In addition, Carvalho et al.^7^ observed 50% sensitivity and 99.17% specificity for rSm200 recombinant protein. Our data support the use of peptides in low endemicity areas and in the prepatent stage of *S. mansoni* infection. As demonstrated in the results shown in Figure 8, the reactivity of the P1425 peptide for detecting the infection is specific and sensitive, which deserves further studies.

The generation of monoclonal antibodies specific to *S. mansoni* antigens has also been a strategy to diagnosis, prevention, and control of the infection.^33^ In the present study, we successfully produced mAbs against the peptide P1425 from *S. mansoni* SmATPDase1, and they were able to bind to the enzyme in immunological assays. SmATPDase1 is a strong antigen candidate for use in immunoassays since it is abundant in the tegument^10^ and may be released into the circulation by the renewal of *Schistosoma* cell structures. Specific antibodies against this protein, like those demonstrated here, can be employed to identify active infection by capture of antigens present in serum or urine of infected patients, and these applications will be explored in further investigations.

## Conclusions

Specific antigens can be applied in indirect tests based on antibodies, improving the detection of *Schistosoma* infection. Evaluating the need for new antigens to diagnose schistosomiasis and the important role that SmATPDase1 plays in *S. mansoni*, we demonstrated by in silico and in vivo approaches that peptides from this protein are potential biomarkers for schistosomiasis diagnosis. We successfully produced anti-peptide antibodies and P1425-mAbs, which could be useful tools for the detection of circulating SmATPDase1. Additionally, peptide P1425 demonstrated robust performance in detecting antibodies, even in low-intensity *S. mansoni* infections, with substantial seropositivity that highlights its capability to distinguish between infected and uninfected mice at an early stage. These findings confirm its valuable potential for early and sensitive diagnosis.

## Materials and Methods

### In Silico Analysis

Amino acid sequences from *S. mansoni*, *S. hematobium,* and *Schistosoma hematobium* ATP diphosphohydrolases and mammalian NTPDases 1–8 were obtained from Universal Protein Resource (https://www.uniprot.org/) and are identified (ID) in figures.

Multiple alignments were performed using Clustal Omega software (https://www.ebi.ac.uk/Tools/msa/clustalo/) with previously acquired amino acid sequences.^21^ To evaluate the presence of peptide sequences in other organisms and therefore reduce cross-reactivity possibilities, search using BLAST (Basic Local Alignment Search Tool; https://www.uniprot.org/blast/) was carried out.

Theoretical physicochemical properties were assessed for the peptides, such as molecular weight, pI, and instability index using the ProtParam online server (https://www.web.expasy.org/protparam/).^22^

A three-dimensional structure of ATP diphosphohydrolase 1 (SmATPDase1) from *S. mansoni* was modeled using PDB 3CJ9 and PDB 3ZX3 X-ray diffraction structure as a template, as described by Nunes et al.^23^

To determine whether the peptides selected are immune cell epitopes, B-cell epitope prediction was carried out by the ABCPred server (https://webs.iiitd.edu.in/raghava/abcpred/)^24^ submitting the SmATPDase1 integral sequence. Applying server thresholds of 0.75 and 14-mer length, epitopes were evaluated. For T-cell epitope prediction, peptides were submitted to the IEDB analysis resource consensus tool.^25,26^

### Synthesis of Peptides

The peptides KDVAKI (P1425), KKPWKDVAKITKLS (P1730), and KDVAKIKDVAKIKDVAKI (P1956) belonging to the SmATPDase1 sequence were synthesized and purified as previously described.^27^

### Experimental Infection and Schistosoma Antigens

Female Swiss mice were infected subcutaneously with one hundred *S. mansoni* cercariae/animal (BH strain). After 55 days, sera samples from healthy (*n* = 12) and infected mice (*n* = 36) were obtained by centrifugation and stored at −20 °C.

For the low parasite burden group, female Swiss mice were also infected subcutaneously with 25 cercariae. The group was subdivided, and sera were collected on different days post-infection (dpi). For the assay in the prepatent period and low parasite burden, sera obtained from 10th to 25th dpi (*n* = 20) and sera from the 35th to 55th dpi (*n* = 15) were used, respectively. At the end of the infection period, adult worms were recovered by perfusion.^28^ They were washed and stored in Tris–HCl buffer with protease inhibitors. Homogenates were obtained by cell disruption cycles in liquid nitrogen.

For tegument isolation, Roberts et al.’s^29^ method was adapted. Adult worms stored in Tris–HCl buffer were disrupted for 15 s on the test tube shaker. The supernatant was centrifuged at 100,000*g* for 1 h, and pellet was resuspended in Tris–HCl buffer plus protease inhibitors. Soluble adult worm antigen (SWAP) was obtained by mechanical grinding, centrifugation, and dialysis process according to Grenfell et al.^30^ Antigens were stored at −20 °C. Protein concentration was determined by the Lowry method.^31^

### Polyclonal Antibodies and Western Blot

To obtain the anti-peptide sera, female Swiss mice received three doses (10 μg) of the peptide P1425 or P1730 by an intraperitoneal route, with an interval of 7 days. The first inoculum was emulsified in complete Freund’s adjuvant and the others with Freund’s incomplete adjuvant. Immune serum samples were obtained after 7 days of the last inoculum and stored at −20 °C.

Aliquots (100 μg of total protein) of adult worm homogenate, tegument, and SWAP preparations were solubilized in gel loading buffer, heated for 5 min at 100 °C, and applied on 10% polyacrylamide gel (Mini-Protean TGX Gels, BioRad) using molecular weight standard as reference.^32^ The run was performed for about 2 h at 100 V in the electrophoresis system (Mini Protean III, BioRad). Next, the gel was electroblotted onto the nitrocellulose membrane (0.45 μm; GE Healthcare, Germany), blocked with PBS-1% casein, and developed with anti-P1425 or anti-P1730 polyclonal antibodies at dilution 1:200. Immunoreactivity was revealed by chemiluminescence using peroxidase-coupled antimouse IgG secondary antibodies at 1:1000 dilution and luminol as the substrate, followed by exposure to X-ray films (ECL Western Blotting System, GE Healthcare Life Sciences) according to the manufacturer’s instructions.

### Enzyme-Linked Immunosorbent Assay (ELISA)

Microtiter plates (Costar 3590, Corning Inc., USA) were coated with 1 μg/mL of peptides in carbonate-bicarbonate buffer, pH 9.6 for 15 h at 4 °C. Plates were washed with phosphate buffer saline-0.05% Tween 20 (PBST) and blocked with PBS-1% casein for 1 h at room temperature. Serum samples from immunized mice diluted 1:20 to 1:160 or infected mice at a dilution 1:200 were added and incubated at room temperature for 1 h. The antibodies were detected by peroxidase-conjugated antimouse diluted at 1:10,000 and colorimetric reaction developed with OPD (*o*-phenylenediamine dihydrochloride) and H_2_O_2_. Reaction was stopped by adding 4NH_2_SO_4_, and the results were read at 492 nm in a microplate reader (Molecular Devices Corp). Reaction was stopped by adding 4 N H_2_SO_4_ and read at 492 nm in a microplate reader (Molecular Devices Corp).

### Immunofluorescence Assay

Cercariae fixed in 10% formaldehyde were permeabilized with 0.2% Triton X-100 and blocked with 1% BSA-PBS (phosphate buffer) for 1 h. For immunostaining, cercariae were incubated with anti-P1425 or anti-P1730 polyclonal antibodies overnight at 4 °C (dilution 1:100). Antimouse secondary antibodies conjugated to Alexa Fluor 488 (1:1000) were used as secondary antibodies (Santa Cruz Biotechnology, CA, USA). Cell nuclei were stained with DAPI (4′,6-diamidino-2-phenylindole dihydrochloride) at 1 μg/mL. Slides were analyzed by a fluorescence microscope (Olympus BX53), using CellsSens Dimension software.

### Production of mAbs

Monoclonal antibodies against the P1425 peptide were produced according to Grenfell et al.^33^ Clones were selected by ELISA. Briefly, 5 μg/mL of the specific peptide diluted in carbonate-bicarbonate buffer (pH, 9.6) was used for plate coating. Supernatant (100 μL) from hybridome cultures was added after blocking the plates with 2.5% milk proteins. Specific mAbs conjugated to peroxidase was used for isotypes’ determination (IgG, IgG1, IgG2a, IgG3, IgM), and plates were revealed after 10 min by incubation with 3,3′,5,5′-Tetramethylbenzidine (TMB) and read at 450 nm in a microplate reader (Thermo Scientific Multiskan FC). Positive reaction was found for the IgG1 isotype. After clone selection and mAbs purification, SDS-PAGE was performed in order to confirm the quality of the produced mAbs.

### Specificity and Affinity Assay

To evaluate mAbs affinity, microtitration plates (Costar^,^ Corning In., NY, USA) were coated overnight at 4 °C with BSA or worm’s tegument diluted at 1 μg/mL in carbonate-bicarbonate buffer, pH 9.6. Anti-peptide IgG1 mAbs were tested in duplicate, diluted 1:50. Additionally, a second ELISA was performed using tegument against pooled sera from C57BL/6 healthy mice or anti-P1425 mAbs (1:50). The reactivity was evaluated according to the Enzyme-linked Immunosorbent Assay (ELISA) Section.

Western blot assays were developed to verify the specificity of the mAbs produced. Tegument or SWAP samples were incubated with anti-P1425 IgG1 mAbs (1:200) and anti-IgG1 antibodies coupled to peroxidase (1:1000), and reaction was detected by chemiluminescence according to the Polyclonal antibodies and Western Blot Section.

### Statistical Analysis

The IgG reactivity was presented as the mean ± SD of OD or EU. Cutoff values were calculated as the mean of the control group plus twice the SD. The statistical significance of differences was determined by Student’s *t*-test and one-way analysis of variance, using GraphPad Prism 5.0 software. ROC curves, sensitivity, and specificity parameters were performed using the MedCalc program.^34^

## Notes

Ethics statement: The in vivo assays were conducted in accordance with Guide for the care and use of laboratory animals approved by the Ethics Committee on the Use of Experimental Animal (CEUA-FIOCRUZ) number LW-21/16.

## References

[ref1] Colley D. G.; Bustinduy A. L.; Secor W. E.; King C. H. Human schistosomiasis. Lancet 2014, 383 (9936), 2253–2264. 10.1016/S0140-6736(13)61949-2. 24698483 PMC4672382

[ref2] LoVerde P. T. Schistosomiasis. In Digenetic tremadodes; Toledo R., Fried B., Eds.; Advances in Experimental Medicine and Biology; Springer, 2019; Vol. 1154, pp 45–70. 10.1007/978-3-030-18616-6_3. 31297759

[ref3] Kittur N.; Castleman J. D.; Campbell C. H.; King C. H.; Colley D. G. Comparison of Schistosoma mansoni prevalence and intensity of infection, as determined by the Circulating Cathodic Antigen urine assay or by the Kato-Katz fecal assay: a systematic review. Am. J. Trop Med. Hyg 2016, 94 (3), 605–610. 10.4269/ajtmh.15-0725. 26755565 PMC4775897

[ref4] Tabios I. K. B.; Sato M. O.; Tantengco O. A. G.; Fornillos R. J. C.; Kirinoki M.; Sato M.; Rojo R. D.; Fontanilla I. K. C.; Chigusa Y.; Medina P. M. B.; Kikuchi M.; Leonardo L. R. Diagnostic performance of parasitological, immunological, molecular, and ultrasonographic tests in diagnosing intestinal schistosomiasis in fieldworkers from endemic municipalities in the Philippines. Front Immunol 2022, 13, 899311 10.3389/fimmu.2022.899311. 35774791 PMC9237846

[ref5] Mohammed H.; Landeryou T.; Chernet M.; Liyew E. F.; Wulataw Y.; Getachew B.; Difabachew H.; Phillips A.; Maddren R.; Ower A.; Mekete K.; Belay H.; Endrias T.; Anjulo U.; Tasew G.; Anderson R.; Tollera G.; Abate E. Comparing the accuracy of two diagnostic methods for detection of light Schistosoma haematobium infection in an elimination setting in Wolaita Zone, South Western Ethiopia. PLoS One 2022, 17 (4), e0267378 10.1371/journal.pone.0267378. 35486627 PMC9053789

[ref6] Doenhoff M. J.; Chiodini P. L.; Hamilton J. V. Specific and sensitive diagnosis of schistosome infection: can it be done with antibodies?. Trends Parasitol 2004, 20 (1), 35–39. 10.1016/j.pt.2003.10.019. 14700588

[ref7] Carvalho G. B. F.; Resende D. M.; Siqueira L. M. V.; Lopes M. D.; Lopes D. O.; Coelho P. M. Z.; Teixeira-Carvalho A.; Ruiz J. C.; Fonseca C. T.; Fonseca C. T. Selecting targets for the diagnosis of Schistosoma mansoni infection: An integrative approach using multi-omic and immunoinformatics data. PLoS One 2017, 12 (8), e0182299 10.1371/journal.pone.0182299. 28817585 PMC5560627

[ref8] Silva-Moraes V.; Shollenberger L. M.; Castro-Borges W.; Rabello A. L. T.; Harn D. A.; Medeiros L. C. S.; Jeremias W. d. J.; Siqueira L. M. V.; Pereira C. S. S.; Pedrosa M. L. C.; Almeida N. B. F.; Almeida A.; Lambertucci J. R.; Carneiro N. F. d. F.; Coelho P. M. Z.; Grenfell R. F. Q.; Grenfell R. F. Q. Serological proteomic screening and evaluation of a recombinant egg antigen for the diagnosis of low-intensity Schistosoma mansoni infections in endemic area in Brazil. PLoS Negl Trop Dis 2019, 13 (3), e0006974 10.1371/journal.pntd.0006974. 30870412 PMC6472831

[ref9] Pearson M. S.; Loukas A.; Sotillo J. Proteomic analysis of Schistosoma mansoni tegumental proteins. Methods Mol. Biol. 2020, 2151, 85–92. 10.1007/978-1-0716-0635-3_8. 32451998

[ref10] Castro-Borges W.; Simpson D. M.; Dowle A.; Curwen R. S.; Thomas-Oates J.; Beynon R. J.; Wilson R. A.; Wilson R. A. Abundance of tegument surface proteins in the human blood fluke Schistosoma mansoni determined by QconCAT proteomics. J. Proteomics. 2011, 74 (9), 1519–1533. 10.1016/j.jprot.2011.06.011. 21704203

[ref11] Skelly P. J.; Wilson R. A. Making sense of the schistosome surface. Adv. Parasitol 2006, 63, 185–284. 10.1016/S0065-308X(06)63003-0. 17134654

[ref12] Da’dara A. A.; Bhardwaj R.; Ali Y. B.; Skelly P. J.; Skelly P. J. Schistosome tegumental ecto-apyrase (SmATPDase1) degrades exogenous pro-inflammatory and pro-thrombotic nucleotides. PeerJ 2014, 2, e316 10.7717/peerj.316. 24711968 PMC3970803

[ref13] Knowles A. F. The GDA1_CD39 superfamily: NTPDases with diverse functions. Purinergic Signal 2011, 7 (1), 21–45. 10.1007/s11302-010-9214-7. 21484095 PMC3083126

[ref14] Zimmermann H. History of ectonucleotidases and their role in purinergic signaling. Biochem. Pharmacol. 2021, 187, 114322 10.1016/j.bcp.2020.114322. 33161020

[ref15] Paes-Vieira L.; Gomes-Vieira A. L.; Meyer-Fernandes J. R. E-NTPDases: Possible Roles on Host-Parasite Interactions and Therapeutic Opportunities. Front. Cell. Infect. Microbiol. 2021, 11, 769922 10.3389/fcimb.2021.769922. 34858878 PMC8630654

[ref16] Carvalho L. S. A.; Alves I. J.; Junqueira L. R.; Silva L. M.; Riani L. R.; Pinto P. d. F.; Filho S.; Alves A. ATP-diphosphohydrolases in parasites: localization, functions and recent developments in drug discovery. Curr. Protein Pept. Sci. 2019, 20 (9), 873–884. 10.2174/1389203720666190704152827. 31272352

[ref17] Vasconcelos E. G.; Nascimento P. S.; Meirelles M. N.; Verjovski-Almeida S.; Ferreira S. T. Characterization and localization of an ATP-diphosphohydrolase on the external surface of the tegument of Schistosoma mansoni. Mol. Biochem. Parasitol. 1993, 58 (2), 205–214. 10.1016/0166-6851(93)90042-v. 8479445

[ref18] DeMarco R.; Kowaltowski A. T.; Mortara R.; Verjovski-Almeida S. Molecular characterization and immunolocalization of Schistosoma mansoni ATP-diphosphohydrolase. Biochem. Biophys. Res. Commun. 2003, 307 (4), 831–838. 10.1016/s0006-291x(03)01268-3. 12878186

[ref19] Faria Pinto P.; Meirelles M. N. L.; Lenzi H. L.; Mota E. M.; Penido M. L. O.; Coelho P. M. Z.; Vasconcelos E. G. ATP diphosphohydrolase from Schistosoma mansoni egg: characterization of a new antigen. Parasitology 2004, 129 (Pt1), 51–57. 10.1017/S0031182004005244. 15267111

[ref20] Levano-Garcia J.; Mortara R. A.; Verjovski-Almeida S.; DeMarco R. Characterization of Schistosoma mansoni ATPDase2 gene, a novel apyrase family member. Biochem. Biophys. Res. Commun. 2007, 352 (2), 384–389. 10.1016/j.bbrc.2006.11.023. 17113569

[ref21] Sievers F.; Higgins D. G. Clustal Omega for making accurate alignments of many protein sequences. Protein Sci. 2018, 27 (1), 135–145. 10.1002/pro.3290. 28884485 PMC5734385

[ref22] Wilkins M. R.; Gasteiger E.; Bairoch A.; Sanchez J. C.; Williams K. L.; Appel R. D.; Hochstrasser D. F. Protein identification and analysis tools in the ExPASy server. Methods Mol. Biol. 1999, 112, 531–552. 10.1385/1-59259-584-7:531. 10027275

[ref23] Nunes V. S.; Vasconcelos E. G.; Faria-Pinto P.; Borges C. C. H.; Capriles P. V. S. Z. Structural comparative analysis of Ecto-NTPDase models from S. mansoni and H. sapiens. In Bioinformatics Research and Applications ISBRA; Harrison R., Li Y., Mandoiu I., Eds.; Springer: Cham, 2015; Vol. 9096, pp 247–259. 10.1007/978-3-319-19048-8_21.

[ref24] Saha S.; Raghava G. P. S. Prediction of continuous B-cell epitopes in an antigen using recurrent neural network. Proteins 2006, 65, 40–48. 10.1002/prot.21078. 16894596

[ref25] Wang P.; Sidney J.; Dow C.; Mothé B.; Sette A.; Peters B. A systematic assessment of MHC class II peptide binding predictions and evaluation of a consensus approach. PLoS Comput. Biol. 2008, 4 (4), e1000048 10.1371/journal.pcbi.1000048. 18389056 PMC2267221

[ref26] Wang P.; Sidney J.; Kim Y.; Sette A.; Lund O.; Nielsen M.; Peters B. Peptide binding predictions for HLA DR, DP and DQ molecules. BMC Bioinf. 2010, 11, 568 10.1186/1471-2105-11-568. PMC2998531 21092157

[ref27] Nakaie C. R.; Oliveira E.; Vicente E. F.; Jubilut G. N.; Souza S. E. G.; Marchetto R.; Cilli E. M. Solid-phase Peptide Synthesis in Highly Loaded Conditions. Bioorg. Chem. 2011, 39 (2), 101–109. 10.1016/j.bioorg.2011.01.001. 21353284

[ref28] Smithers S. R.; Terry R. J. The infection of laboratory hosts with cercariae of Schistosoma mansoni and the recovery of the adult worms. Parasitology 1965, 55 (4), 695–700. 10.1017/S0031182000086248. 4957633

[ref29] Roberts S. M.; MacGregor A. N.; Vojvodic M.; Wells E.; Crabtree J. E.; Wilson R. A. Tegument surface membranes of adult Schistosoma mansoni-: development of a method for their isolation. Mol. Biochem. Parasitol. 1983, 9 (2), 105–127. 10.1016/0166-6851(83)90104-4. 6669162

[ref30] Grenfell R. F. Q.; Martins W. H.; Silva-Moraes V.; Barata S. V.-B.; Ribeiro E. G.; Oliveira E.; Coelho P. M. Z. Antigens of worms and eggs showed a differentiated detection of specific IgG according to the time of Schistosoma mansoni infection in mice. Rev. Soc. Bras Med. 2012, 45 (4), 505–509. 10.1590/S0037-86822012000400018. 22930047

[ref31] Lowry O. H.; Rosebrough N. J.; Farr A. L.; Randall R. J. Protein measurement with the Folin phenol reagent. J. Biol. Chem. 1951, 193, 265–275. 10.1016/S0021-9258(19)52451-6. 14907713

[ref32] Laemmli U. K. Cleavage of structural proteins during the assembly of the head of bacteriophages T4. Nature 1970, 227 (5259), 680–685. 10.1038/227680a0. 5432063

[ref33] Grenfell R. F. Q.; Coelho P. M. Z.; Taboada D.; de Mattos A. C. A.; Davis R.; Harn D. A.; Harn D. A. Newly established monoclonal antibody diagnostic assays for Schistosoma mansoni direct detection in areas of low endemicity. PLoS One 2014, 9 (1), e87777 10.1371/journal.pone.0087777. 24498191 PMC3909226

[ref34] Schoonjans F.; Zalata A.; Depuydt C. E.; Comhaire F. H. MedCalc: a new computer program for medical statistics. Comput. Methods Programs Biomed 1995, 48 (3), 257–262. 10.1016/0169-2607(95)01703-8. 8925653

[ref35] Emídio N. B.; Gusmão M. A. d. N.; Castro-Borges W.; Nakaie C. R.; Vasconcelos E. G.; Pinto P. d. F. Priscila. Identification of a linear IgE inducing epitope on the SmATPDase1 surface. Acta Biochim Biophys Sin. 2017, 49 (6), 564–566. 10.1093/abbs/gmx031. 28398461

[ref36] Fonseca C. T.; Carvalho G.^nia B. F.; Alves C. C.; Melo T. T. Schistosoma tegument proteins in vaccine and diagnosis development: an update. J. Parasitol Res. 2012, 2012, 541268 10.1155/2012/541268. 23125917 PMC3483795

[ref37] Faria-Pinto P.; Rezende-Soares F. A.; Molica A. M.; Montesano M. A.; Marques M. J.; Rocha M. O. C.; Gomes J. A. S.; Enk M. J.; Correa-Oliveira R.; Coelho P. M. Z.; Neto S. M.; Franco O. L.; Vasconcelos E. G. Mapping of the conserved antigenic domains shared between potato apyrase and parasite ATP diphosphohydrolases: potential application in human parasitic diseases. Parasitology 2008, 135 (4), 943–953. 10.1017/S0031182008004538. 18598576

[ref38] Maia A. C. R. G.; Detoni M. L.; Porcino G. N.; Soares T. V.; do Nascimento Gusmão M. A.; Fessel M. R.; Marques M. J.; Souza M. A.; Coelho P. M. Z.; Estanislau J. A. S. G.; da Costa Rocha M. O.; de Oliveira Santos M.; Faria-Pinto P.; Vasconcelos E. G. Occurrence of a conserved domain in ATP diphosphohydrolases from pathogenic organisms associated to antigenicity in human parasitic diseases. Dev. Comp. Immunol. 2011, 35 (10), 1059–1067. 10.1016/j.dci.2011.03.026. 21527274

[ref39] Yagi M.; Bang G.; Tougan T.; Palacpac N. M. Q.; Arisue N.; Aoshi T.; Matsumoto Y.; Ishii K. J.; Egwang T. G.; Druilhe P.; Horii T. Protective epitopes of the Plasmodium falciparum SERA5 malaria vaccine reside in intrinsically unstructured n-terminal repetitive sequences. PLoS One 2014, 9 (6), e98460 10.1371/journal.pone.0098460. 24886718 PMC4041889

[ref40] Sanches R. C. O.; Tiwari S.; Ferreira L. C. G.; Oliveira F. M.; Lopes M. D.; Passos M. J. F.; Maia E. H. B.; Taranto A. G.; Kato R.; Azevedo V. A. C.; Lopes D. O. Immunoinformatics design of multi-epitope peptide-based vaccine against Schistosoma mansoni using transmembrane proteins as a target. Front. Immunol. 2021, 12, 621706 10.3389/fimmu.2021.621706. 33737928 PMC7961083

[ref41] Rehman A.; Ahmad S.; Shahid F.; Albutti A.; Alwashmi A. S. S.; Aljasir M. A.; Alhumeed N.; Qasim M.; Ashfaq U. A.; Tahir ul Qamar M. Integrated core proteomics, subtractive proteomics, and immunoinformatics investigation to unveil a potential multi-epitope vaccine against schistosomiasis. Vaccines 2021, 9 (6), 658 10.3390/vaccines9060658. 34208663 PMC8235758

[ref42] Jensen K. K.; Andreatta M.; Marcatili P.; Buus S. .; Greenbaum J. A.; Yan Z.; Sette A.; Peters B.; Nielsen M. Improved methods for predicting peptide binding affinity to MHC class II molecules. Immunology 2018, 154 (3), 394–406. 10.1111/imm.12889. 29315598 PMC6002223

[ref43] Vasconcelos E.; Ferreira S. T.; Carvalho T.; Souza W.; Kettlun A. M.; Mancilla A. M.; Valenzuela M. A.; Verjovski-Almeida S. Partial purification and immunohistochemical localization of ATP diphosphohydrolase from Schistosoma mansoni. Immunological cross-reactivities with potato apyrase and Toxoplasma gondii nucleoside triphosphate hydrolase. J. Biol. Chem. 1996, 271 (36), 22139–22145. 10.1074/jbc.271.36.22139. 8703025

[ref44] Mendes R. G. P. R.; Gusmão M. A. d. N.; Maia A. C. R. G.; Detoni M. d. L.; Porcino G. N.; Soares T. V.; Juliano M. A.; Juliano L.; Coelho P. M. Z.; Lenzi H. L.; Faria-Pinto P.; Vasconcelos E. G.; Priscila F.-P.; Vasconcelos E. G. Immunostimulatory property of a synthetic peptide belonging to the soluble ATP diphosphohydro-lase isoform (SmATPDase 2) and immunolocalisation of this protein in the Schistosoma mansoni egg. Mem. Inst. Oswaldo Cruz 2011, 106, 808–813. 10.1590/S0074-02762011000700005. 22124552

[ref45] Porcino G. N.; Carvalho-Campos C.; Maia A. C. R. G.; Detoni M. L.; Faria-Pinto P.; Coimbra E. S.; Marques M. J.; Juliano M. A.; Juliano L.; Diniz V. Á.; Corte-Real S.; Vasconcelos E. G. Leishmania (Viannia) braziliensis nucleoside triphosphate diphosphohydrolase (NTPDase 1): Localization and in vitro inhibition of promastigotes growth by polyclonal antibodies. Exp. Parasitol. 2012, 132 (2), 293–299. 10.1016/j.exppara.2012.08.009. 22921497

[ref46] Maia A. C. R. G.; Porcino G. N.; Detoni M. de L.; Emídio N. B.; Marconato D. G.; Faria-Pinto P.; Fessel M. R.; Reis A. B.; Juliano L.; Juliano M. A.; Marques M. J.; Vasconcelos E. G. An antigenic domain within a catalytically active Leishmania infantum nucleoside triphosphate diphosphohydrolase (NTPDase 1) is a target of inhibitory antibodies. Parasitol Int. 2013, 62 (1), 44–52. 10.1016/j.parint.2012.09.004. 22995148

[ref47] Graeff-Teixeira C.; Favero V.; Souza R. P.; Pascoal V. F.; Bittencourt Hélio R.; Fukushige M.; Geiger S. M.; Negrão-Corrêa D. Use of Schistosoma mansoni soluble egg antigen (SEA) for antibody detection and diagnosis of schistosomiasis: The need for improved accuracy evaluations of diagnostic tools. Acta Trop. 2021, 215, 105800 10.1016/j.actatropica.2020.105800. 33352167

[ref48] Oyeyemi O. T.; Corsini C. A.; Gonçalves G.; de Castro Borges W.; Grenfell R. F. Q.; Queiroz R. F. Evaluation of schistosomula crude antigen (SCA) as a diagnostic tool for Schistosoma mansoni in low endemic human population. Sci. Rep. 2021, 11 (1), 10530 10.1038/s41598-021-89929-3. 34006964 PMC8131376

[ref49] Grenfell R.; Martins W.; Silva-Moraes V.; Araujo N.; Oliveira E.; Fonseca C.; Coelho P. M. Z. The schistosomula tegument antigen as a potential candidate for the early serological diagnosis of schistosomiasis mansoni. Rev. Inst Med. Trop São Paulo 2013, 55 (2), 75–78. 10.1590/S0036-46652013000200002. 23563758

